# Helping Clinicians Conceptualise Behavioural Insomnia in Children: Development of the Manifestations and Vulnerabilities of Behavioural Insomnia in Childhood Scale (MAVBICS)

**DOI:** 10.1007/s10578-023-01606-w

**Published:** 2023-10-05

**Authors:** Caroline L. Donovan, Laura Uhlmann, Amy Shiels

**Affiliations:** 1 School of Applied Psychology, Griffith University, 176 Messines Ridge Rd, Mt Gravatt, Brisbane, QLD 4122 Australia; 2 Griffith University Centre for Mental Health, Griffith University, Brisbane and Gold Coast, QLD Australia

**Keywords:** Child, Sleep, Assessment, Measure, Insomnia

## Abstract

This paper outlines the development and psychometric evaluation of the Manifestations and Vulnerabilities of Behavioural Insomnia in Childhood Scale (MAVBICS), an instrument intended to assess the manifestations of, and factors underpinning, child behavioural insomnia. The MAVBICS comprises two sections: a more general sleep and bedtime information section (Section 1), and a psychometric measure of six theoretically derived factors that underlie, contribute to, and are manifestations of, child sleep problems (Section 2), that is the focus of this research. Study 1 comprised an exploratory factor analysis of Section 2 items (EFA; n = 328 parents of children aged 3–12 years), with a final 25 items found to load highly onto 6 factors; Sleep Maintenance Problems (4 items, α = 0.88), Co-Sleeping Behaviours (4 items, α = 0.93), Bedtime Routines (5 items, α = 0.82), Bedtime Resistance (5 items, α = 0.88), Bedtime Worries (3 items, α = 0.85) and Bedtime Fears (4 items, α = 0.86). Study 2 comprised a confirmatory factor analysis (CFA) of Section 2 items and tests of convergent validity (n = 313), with results confirming the factor structure and providing evidence for convergent validity through correlations in expected directions between MAVBICS scores and other sleep, anxiety and behaviour measures. Study 3 tested the test–retest reliability of Section 2 items (n = 53), and found support for the temporal stability of the MAVBICS over a 2-week period. Overall, the results provide strong preliminary evidence for the validity of the MAVBICS total score and its subscales, although the Bedtime Routines subscale may be less useful.

**Supplementary Information:**

The online version contains supplementary material available at 10.1007/s10578-023-01606-w.

## Introduction

Sleep problems in childhood are qualitatively different to those experienced in adolescence and adulthood. Children may experience a variety of sleep problems including respiratory disorders (e.g., sleep apnoea), parasomnias (e.g., nightmares, night terrors, sleep walking, sleep paralysis, and confusional arousals), and most commonly, behavioural sleep problems (Melzter 2021). The International Classification for Sleep Disorders, 3rd Edition (ICSD-3; [1], refers to child behavioural sleep problems as ‘Behavioural Insomnia of Childhood’, a disorder whereby children exhibit difficulty initiating and maintaining sleep, exhibit bedtime resistance, and / or have difficulty sleeping independently or without parental assistance. Behavioural insomnia afflicts 10–30% of children [2], is unlikely to remit if left untreated [3], and is associated with numerous problematic consequences both in the short- and long-term. Indeed, it has been linked cross sectionally and longitudinally to impaired academic performance, reduced cognitive functioning, anxiety related disorders, depressive disorders, schizophrenia spectrum and bipolar related disorders, behavioural disturbances, and obesity, as well as maternal depression and parental and family related stress [4–6].

Given the high prevalence and deleterious outcomes of behavioural insomnia in children, early intervention is vital. However, to successfully treat behavioural insomnia, clinicians must first (a) obtain specific information around the child’s sleep patterns and problems and (b) conceptualise the child’s difficulties by determining factors underpinning and contributing to a child’s particular sleep problem(s). With respect to specific information around a child’s sleep patterns and problems, information regarding sleep and wake times, sleep onset latency (the time it takes a child to fall asleep after being put to bed), sleep duration, number and frequency of daytime naps, and the number and frequency of night-time awakenings, is required. Such information allows the clinician to determine the type of behavioural sleep problem a child demonstrates (i.e., problems with sleep onset, sleep duration, sleep maintenance, and/or developmentally inappropriate sleep and wake times). In addition, information pertaining to the existence of parasomnias or respiratory problems is essential, as such difficulties require a treatment approach that is different to that taken for behavioural sleep problems.

In terms of conceptualising a child’s behavioural sleep problem(s), clinicians require information on a range of factors that may represent manifestations of, or may underly and/or contribute to, the child’s particular sleep difficulty. The pathophysiology of sleep related problems can be complex—especially among paediatric populations, with a number of potential child and parent factors at play. Parent factors include poor implementation of bedtime routines, allowance of poor sleep hygiene, and engagement in co-sleeping. Bedtime routines implemented by parents can inadvertently contribute to child sleep problems if implemented inconsistently (e.g., varied timing of pre-bedtime activities), incorrectly (e.g., placing a child to bed too early or late), or not at all [7]. In particular, a poor bedtime routine disrupts sleep onset and therefore reduces sleep duration, as the parent struggles to get the child to bed at the desired time. Similarly, inadequate sleep hygiene behaviours such as engaging in alerting pre-bedtime activities, child consumption of caffeinated food and drinks, and non-sleep conducive environments, have all been linked with sleep impairment in childhood by making it physiologically difficult for a child to sleep [4, 8]. Finally, when parents and children co-sleep, the child becomes dependent on the parent to feel safe at night and to initiate and maintain sleep, disallowing the opportunity for the child to learn to fall asleep alone [7, 9].

With respect to child factors that may contribute to behavioural sleep problems, oppositional behaviour at bedtime, worry at bedtime, bedtime fears, and an inability to return to sleep unaided, are manifestations of behavioural insomnia itself (as described in the ICSD-(3) and/or may underlie or contribute to, a child’s sleep problem. Both oppositional behaviour and anxiety have shown strong associations with sleep problems in children [10–12]. Oppositional behaviour at bedtime is also referred to as bedtime resistance and includes a range of behaviours such as crying, tantruming, refusal to bathe and put on pyjamas, refusal to get into bed and stay in bed, and running away from parents. Anxiety and sleep problems have a similarly strong association in children, manifesting as worry at bedtime (e.g., about upcoming or past events and interactions) and/or specific bedtime fears (such as separation concerns, fear of the dark, monsters, burglars etc.). Both anxiety and bedtime resistance serve to physiologically and cognitively arouse the child and/or delay them getting into bed, thus delaying sleep onset and reducing sleep duration [13]. Finally, inability to return to sleep unaided may also be a manifestation of and/or may underpin sleep problems in children. All people, including children, ‘wake’ during the night, but most can put themselves back to sleep immediately, often without realising they have woken [14, 15]. However, some children exhibit difficulty putting themselves back to sleep (commonly referred to as sleep maintenance problems), which contributes to an increase in the duration of night awakenings and a reduction in overall sleep duration [14, 15].

It is clear from the above discussion that the same sleep patterns or problems (e.g., difficulty with sleep onset, maintenance, or duration) may be the result of different underlying or contributing factors (co-sleeping, bedtime resistance, sleep hygiene, bedtime fears or worries, problems with sleep maintenance, and/or poor bedtime routines), each of which require different cognitive behavioural treatment strategies. The development of a comprehensive, multidimensional measure that assesses the manifestations of behavioural insomnia in children, as well as the factors underpinning and contributing to it, would therefore be of significant value to clinicians as it would assist with conceptualisation and treatment planning. To date, although numerous scales in the literature purport to measure child sleep problems, they are limited by poor psychometric properties, lack of psychometric evaluation, and/or reduced assessment scope. Indeed, none of the childhood sleep scales currently available provide a comprehensive *and* valid assessment of the manifestations of, and factors underpinning and contributing to, child behavioural sleep problems.

### Existing Measures of Paediatric Sleep Problems

An initial review by Spruyt and Gozal [16] of scales assessing paediatric sleep problems, found that only two scales (the Sleep Disorders Inventory for Students-Child [17] and the Sleep Disturbance Scale for Children [18]) met all appropriate methodological and psychometric scale development criteria. An examination of the items comprising these scales however, reveals that they assess the physiological and medical aspects of sleep problems in children (e.g., excessive daytime tiredness, breathing difficulties, sleep related movement disorders and parasomnias) rather than the underpinning and contributing factors of behavioural insomnia that are targeted in cognitive behavioural treatment programs. Further, although the Sleep Disturbance Scale for Children includes items assessing issues such as bedtime resistance and anxiety, they are represented by a single item only (e.g., ‘the child goes to bed reluctantly’, ‘the child feels anxious or afraid when falling asleep’), and together comprise a larger factor measuring overall problems with sleep initiation and maintenance [17]. Although these scales are useful for the identification of paediatric sleep problems, they are less useful for providing a comprehensive assessment of the potential factors underpinning such problems and informing targets for treatment.

A recent updated review conducted by Sen and Spruyt [19] reported on an additional 27 novel paediatric sleep tools developed since the initial review. Importantly, only two of the additional new scales met the outlined criteria for adequate development, with neither scale purporting to assess the underpinnings and contributing factors of behavioural paediatric insomnia. Specifically, the SNAKE questionnaire [20] evaluates paediatric sleep issues and conditions associated with severe psychomotor impairment, whilst the Narcolepsy Quality of Life-21 (NARQoL-21; [21] assesses the quality of life of children with narcolepsy. Thus, within the literature there appears to be a lack of well validated psychometric tools developed for the specific purpose of providing a multidimensional assessment of the potential factors underpinning behavioural insomnia in children.

Currently, the most popular measure of child behavioural sleep problems is the Children’s Sleep Habits Questionnaire (CSHQ; [22]. The CSHQ assesses sleep problems and their aetiology via eight domains: bedtime resistance, sleep onset delay, sleep duration, sleep anxiety, night waking, parasomnias, sleep disordered breathing and daytime sleepiness. However, the CSHQ is not without limitations. For example, the bedtime resistance subscale contains items that appear to measure resistance (e.g., ‘struggles at bedtime’), fear (e.g., ‘afraid of sleeping alone’), routine (e.g., ‘goes to bed at the same time’), and co-sleeping (‘needs a parent in the room to sleep’). While it is reasonable to assume that each of these problems could result in, or cause, resistance at bedtime, the extent of resistance is not actually assessed, and the reasons for resistance are not differentiated. Further, while issues such as co-sleeping, bedtime fears, and lack of a bedtime routine constitute important avenues for psychological intervention, a one-item assessment does not allow for the assessment of problem severity, meaning that primary and secondary issues are more difficult to determine, and treatment response is more difficult to assess. Most importantly perhaps, the psychometric properties of the CSHQ have recently come into question. In the review by Spruyt and Gozal [16], it was noted that the questionnaire lacked a number of key methodological and psychometric criteria (i.e., pilot testing, item analyses, factor analyses, confirmatory factor analyses and norm generation). Moreover, in the paper reporting the scale’s development, only one subscale (bedtime resistance) was shown to demonstrate good internal consistency (i.e., Cronbach’s alpha > 0.70; [22]. Thus, despite the popularity of the CSHQ, the brevity and limited psychometric testing of this measure place a ceiling on its clinical and empirical usefulness.

It is clear from the above discussion that a comprehensive, clinically useful, well operationalised and psychometrically validated measure of behavioural sleep problems and the factors known to underlie them in childhood is currently lacking. The overarching aim of the current research therefore, was to develop and psychometrically evaluate across a series of three studies, the Manifestations and Vulnerabilities to Behavioural Insomnia in Childhood Scale (MAVBICS).

As noted above, it is important for clinicians to both obtain specific information around the child’s sleep patterns and problems and to conceptualise the child’s sleep problem(s) by determining factors underpinning and contributing to them. The MAVBICS therefore comprises two sections, (1) a more general sleep and bedtime information section (see supplementary material for a copy of Section 1 of the MAVBICS), and (2) a psychometric measure of six theoretically derived factors that represent manifestations of and/or factors that underlie and contribute to, child sleep problems. In this way, the MAVBICS is similar to other measures developed for both clinical and research purposes such as the Child Behaviour Checklist (CBCL; [23] and the Eating Disorder Inventory (EDI; [24, 25]. Section 2 of the MAVBICS is the focus of the current series of three studies. Study 1 documents the development of Section 2 MAVBICS items and the exploratory factor analysis conducted on the items produced. Study 2 reports on the confirmatory factor analysis of Section 2 of the MAVBICS, and the subsequent testing of its convergent validity. Finally, Study 3 assesses Section 2 of the MAVBICS in terms of its 2-week test–retest reliability. Data was collected from July to October of 2019.

## Study 1

The aim of Study 1 was to develop Section 2 of the MAVBICS, which included 6 factors that, from a cognitive-behavioural perspective, are thought to be manifestations of, and/or to underly or contribute to, sleep problems in children: Bedtime Routines, Bedtime Fears, Bedtime Worries, Bedtime Resistance, Sleep Maintenance Problems, and Co-Sleeping Behaviours. It was hypothesised that the exploratory factor analysis (EFA) of these items would produce 6 factors corresponding to the 6 constructs.

## Study 1 Method

### Participants

The participants were 328 male (3.4%) and female (96%) primary caregivers aged between 18 and 56 years (M = 36.47, *SD* = 5.85), who reported being either the mother (95.4%), father (2.7%), grandparent (0.3%), relative (1.2%) or other caregiver (0.3%) of a child aged between 3 and 12 years. Another 34 participants began the questionnaire but were excluded due to obviously erroneous data entry, failure to consent, or having children outside the selected age range. Supplementary Tables 1 and 2 report detailed demographic information for parents and children respectively. Of the participants, 10.7% were university students, 37.2% reported a bachelor degree as their highest level of education, 46% reported a household income between $100,001 and $200,000, 78.3% were employed full time or part time, and 89.9% were Australian Caucasian. Of the participants in the sample, 55.2% reported having a child aged 3–5 years. Most participants (97.9%) reported having a child without any formal mental health, sleep or developmental diagnosis. Of the sample, 30.2%, 8.2% and 1.8% of the sample believed that their child had a mild, moderate and severe sleep problem respectively.

**Table 1:** Study 1 EFA and Study 2 CFA factor loadings for the MAVBICS items

		Study 1: (*N* = 328) EFA factor loadings						Study 2: (*N = *313) standardised factor loadings					
	Mean (SD)	SM	CS	BRo	BRe	BW	BF	SM	CS	BRo	BRe	BW	BF
1. My child sleeps through the whole night*	2.8 (1.7)	0.83						0.83					
2. My child wakes up during the night and wakes another family member	2.8 (1.6)	0.75						0.87					
3. After falling asleep, my child wakes and calls out	2.2 (1.4)	0.73						0.75					
4. My child repeatedly gets out of their bed at night time	2.2 (1.4)	0.51						0.69					
5. At night, my child sleeps in the same bed as me, or another family member	2.6 (1.8)		0.95						0.85				
6. My child sleeps alone in their bed throughout the night*	2.7 (1.9)		0.89						0.93				
7. My child refuses to sleep in their own room by themselves	2.5 (1.8)		0.82						0.77				
8. My child falls asleep, by themselves, in their own bed*	2.9 (2.0)		0.72						0.72				
9. In our household, there is a set order of activities before bed*	2.2 (1.2)			0.85						0.86			
10. In our household, all caregivers follow the same bedtime routine with my child*	2.2 (1.2)			0.71						0.75			
11. In our household, the activities before my child’s bedtime are the same each night*	2.2 (1.0)			0.69						0.85			
12. My child knows the activities they need to do before bed*	1.8 (1.0)			0.66						0.84			
13. I attempt to put my child to bed at the same time each night (within 15 min)*	1.9 (1.0)			0.55						0.67			
14. My child does things to delay going to bed	3.4 (1.4)				0.84						0.87		
15. My child does not follow instructions bedtime	2.4 (1.2)				0.82						0.73		
16. My child tantrums when asked to do things at bedtime	2.2 (1.2)				0.75						0.66		
17. My child does not want to go to bed	3.2 (1.4)				0.71						0.80		
18. At bedtime, my child makes repeated requests (e.g., for a drink, for food, for a cuddle)	2.8 (1.4)				0.69						0.77		
19. My child worries about many different things when in bed	2.0 (1.2)					0.87						0.87	
20. My child lies awake at night worrying	1.8 (1.0)					0.80						0.92	
21. My child finds it difficult to sleep because they can't stop thinking	2.7 (1.5)					0.69						0.78	
22. My child is scared of monsters, witches, or other scary things at night time	2.2 (1.3)						0.86						0.83
23. My child gets scared at bed/night time	2.1 (1.2)						0.73						0.89
24. My child is frightened of the dark	2.8 (1.6)						0.72						0.68
25. My child needs someone to tell them it's OK at night (e.g., about the dark, being alone, safety, monsters)	2.5 (1.5)						0.61						0.78
Eigenvalues		8.16	2.85	2.59	2.15	1.38	1.23	–	–	–	–	–	–
Item variance explained, %		32.65	11.38	10.35	8.60	5.52	4.91	–	–	–	–	–	–
Cronbach’s α		0.88	0.93	0.82	0.88	0.85	0.86	0.86	0.88	0.92	0.88	0.89	0.89
Mean (standard deviation)		6.01 (5.23)	6.68 (6.75)	5.28 (4.19)	9.00 (5.54)	3.58 (3.31)	5.60 (4.74)	6.54 (4.97)	6.91 (6.14)	5.62 (5.12)	10.11 (5.54)	4.26 (3.89)	5.80 (4.77)
Range		0–20	0–20	0–25	0–25	0–15	0–20	0–20	0–20	0–25	0–25	0–15	0–20

Note in study 1 EFA, all factor loadings below. 20 are suppressed. CFA is based on MLR estimation. See text for explanations of analyses used in Study 1 and Study 2. Asterisked items are reverse scored

*SM* sleep maintenance, *CS* So-Sleeping behaviours, *BRo* bedtime routines, *BRe* bedtime resistance, *BW* bedtime worries, *BF* bedtime fears. Factor structure did not differ based on child age demographics (i.e., 3–6 years versus 7–12 years)

^*^Reverse scored items

**Table 2:** Study 2 Correlations between the MAVBICS subscales, MAVBICS total score, and convergent validity measures

	1	2	3	4	5	6	7	8	9	10	11	12	13	14	15	*Mean*	*SD*
1. MAVBICS Total	1															40.27	19.48
2. MAVBICS BRe	0.57**	1														2.05	0.69
3. MAVBICS CoS	0.66**	0.14*	1													1.73	1.53
4. MAVBICS BF	0.71**	0.36**	.32**	1												1.37	1.14
5. MAVBICS BW	0.60**	0.41**	0.11**	0.55**	1											1.43	1.29
6. MAVBICS SM	0.72**	0.35**	0.42**	0.42**	0.37**	1										1.54	1.13
7. MAVBICS BRo	0.36**	− 0.06	0.20**	− 0.02	− 0.01	0.09	1									1.12	1.03
8. CSHQ Total	0.79**	0.43**	0.60**	0.45**	0.46**	0.67**	0.22**	1								47.00	8.83
9. CSHQ BR	0.67**	0.24**	0.84**	0.36**	0.19**	0.41**	0.22**	0.70**	1							9.06	3.08
10. CSHQ SA	0.69**	0.26**	0.68**	0.59**	0.34**	0.50**	0.04	0.71**	0.80**	1						6.27	2.16
11. CSHQ NW	0.57**	0.25**	0.46**	0.30**	0.21**	0.81**	-0.02	0.62**	0.41**	0.48**	1					4.62	1.80
12. SCAS	0.62**	0.36**	0.28**	0.58**	0.66**	0.36**	0.13	0.50**	0.34**	0.44**	0.24**	1				33.86	20.86
13. PAS	0.56**	0.33**	0.31**	0.61**	0.60**	0.34**	0.11	0.39*	0.39**	0.57**	0.27*	–	1			20.00	15.19
14. SDQ_C_YC	0.36**	0.40**	0.16	0.22*	0.27**	0.33**	0.05	0.43**	0.27*	0.27**	0.34*	–	0.13	1		2.28	1.87
15. SDQ_C	0.31**	0.42**	0.11	0.09	0.21**	0.23**	0.08	0.38**	0.24**	0.17*	0.15*	0.26**	–	–	1	2.92	1.35

*MAVBICS* Manifestations and vulnerabilities to behavioural insomnia in childhood scale, *BRe* bedtime resistance, *CoS* co-sleeping, *BF* bedtime fears, *BW* bedtime worries, *SM* sleep maintenance, *Bro* bedtime routines, *CSHQ* child sleep habits questionnaire, *BR* bedtime resistance, *SD* sleep duration, *SA* sleep anxiety, *NW* night waking, *SCAS* spence childhood anxiety scale, *PAS* preschool anxiety scale, *SD* = standard deviation

– = NA due to different age sample used, Total = composite score

* = *p* < .05, ** = *p* < .01

### Measures

#### Caregiver Demographics

Participants were asked a series of single item demographic questions relating to their gender, age, ethnicity, relationship to the child, relationship status, employment status, highest level of education, parenting status, and annual household income.

#### Child Demographics

Participants were asked a series of demographic questions relating to the child’s gender, age, ethnicity, whether the child resides in one or more residences, and the presence of any formal diagnoses.

#### Parent-Reported Sleep Problems

Participants were asked to rate (irrespective of any formal diagnosis) whether they felt their child suffered with a sleep related problem. Participants indicated their answer on a four-point Likert-type scale, where 0 = no problem, 1 = mild problem, 2 = moderate problem, and 3 = severe problem.

#### The MAVBICS

Items for Section 2 of the MAVBICS were generated from a comprehensive review of the paediatric sleep literature as well as consultation with experts in child sleep and anxiety (clinical and research experts), and a critical review of existing scales. A scale blueprint (i.e., the hypothesized factor structure) was created and served as a framework for item creation [26]. Items were then created specifically to tap each content area (i.e., factor). An initial 44 items were developed to address six key content areas (i.e., Bedtime Routines [5 items], Bedtime Fears [8 items], Bedtime Worries [5 items], Bedtime Resistance and/or Oppositional Bedtime Behaviour [12 items], Sleep Maintenance Problems [7 items] and Co-Sleeping Behaviour [7 items]).

Items were written to avoid double-barrelled questions and complex wording, and included a mix of positively and negatively worded items. Insurance against ambiguity of items and indecisiveness, acquiescence and social desirability were tackled at the item development and expert review stages. The expert panel, consisting of 7 researchers from the USA and Australia, who had published scales or other research in paediatric mental health, were consulted on the six content areas and the appropriateness of items generated for each content area, including item phrasing. Clark and Watson [27] suggest erring on the side of over-inclusiveness when generating initial items, and to rely on the subsequent psychometric analysis to identify weak or unrelated items. For this reason, items that were unanimously identified as being problematic, were deleted at the expert review stage, and other problematic items were flagged for review in the psychometric stages.

The instructions for the scale were as follows; “*Below is a series of statements that describe a range of bedtime and sleep related behaviours your child might demonstrate. We would like you to think about your child and their behaviour over the past week when responding to the questions below. If there was a reason that the last week was unusual (such as your child was unwell or there was a holiday period), respond to the questions based on a typical week. Please think about the following statements in relation to your child and household, and rate how true each of the following statements are, using the following scale:”* The six-point scale ranged from 0 (*never true*), to 5 (*always true*), and subscale items were summed so that higher scores reflected greater sleep related difficulties. Thus, the asterisked items displayed in Table 1 are reverse scored before summing. A total score was obtained by summing the MAVBICS subscale scores.

### Procedure

Approval for the study was granted by the Griffith University Human Ethics Review Committee (HREC: 2019/785). All student participants were recruited through the psychology student subject pool, and all non-students were recruited through the university email call for research volunteers, and via social networking sites (e.g., Facebook). Recruitment material informed potential participants of eligibility criteria for participation (i.e., a primary caregiver of a child aged between 3 and 12 years) and provided a web link that directed them to the online survey hosted on Qualtrics. After clicking on the link, individuals were directed to a downloadable information sheet and consent form that outlined the purpose of the study, participation requirements, and confidentiality. The information sheet informed participants that the research was being conducted to develop a new measure of child sleep related problems, and that they would be asked to answer a series of questions related to child sleep, family life, and personal experiences as a parent. Participants were then instructed to indicate, via radio buttons, that they had read the information and consent forms and consented to participate in the research. Only those who provided informed consent went on to complete the questionnaire battery. All items within the battery were compulsory for participants to complete. Following completion of the questionnaire, participants were invited to enter their name into a draw to win one of ten $50 gift vouchers (non-students) or gain course credit (students) for their participation.

### Data Analytic Procedure

To explore the factor structure of the 44 items, a series of EFAs were conducted specifying principal axis factoring and an oblique (i.e., direct oblimin) rotation using the IBM SPSS Statistics package. Bartlett’s test of sphericity and the Kaiser–Meyer–Olkin measure of sampling adequacy were used to assess the factorability of the items. Overall sampling adequacy can be concluded when Bartlett’s test of Sphericity is significant and the Kaiser–Meyer–Olkin value is greater than 0.60 [28]. In line with best practice, the number of factors retained was determined by parallel analysis, the Kaiser–Guttman criterion (i.e., retention of factors with eigenvalues 1.0), examination of the scree plot, and investigation of the pattern matrix (O'Connor, 2000). Theory and the designed blueprint were also used to guide decision making around the final items to retain after EFA [27, 29].

## Study 1 Results

### Preliminary Analyses

Barlett’s test of sphericity was significant (χ2 = 6053.34, *df* = 378, *p* < 0.001) and the Kaiser–Meyer–Olkin value was 0.90, indicating that the initial 44 MAVBICS items were appropriate for factor analysis. Additionally, all measures of sampling adequacy taken from the diagonal of the anti-image correlation table were =  > 0.80.

### Exploratory Factor Analysis

Prior to exploratory factor analysis (EFA), items were removed if they met two or more of the following criteria: (1) item redundancy or insufficient correlations with other items (i.e., inter-item correlations of *r* > 0.8 or < 0.2 respectively), (2) poor item statistics (i.e., if all response options were not utilized), and (3) age bias (i.e., if a singular item correlated (*r* > 0.35) with the reported age of the parent [26, 29]. As a result, 11 items were excluded from further analyses, leaving 33 items for the EFA.

The first EFA resulted in the extraction of six factors with eigenvalues greater than 1 (i.e., 11.32, 3.33, 2.98, 2.63, 1.59, and 1.42 respectively). Parallel analysis revealed 15 eigenvalues greater than 1, the first six of which (i.e., 1.65, 1.56, 1.49, 1.44, 1.39, and 1.35 respectively) were smaller than those extracted through EFA [30]. Inspection of the scree plot revealed an inflection point between 6 and 7 factors. Thus, results converged on a 6-factor structure. Inspection of the pattern matrix revealed that all factors aligned with the hypothesised blueprint.

Items loading on the six factors were evaluated for deletion against the following criteria; (1) poor factor loadings (i.e., loadings < 0.40) or small communalities (i.e., < 0.40), (2) cross-loadings on two or more factors (i.e., loadings > 0.3 on the second factor), (3) a lack of conceptual/face validity (i.e., if the loading of an item on a factor did not align with theory or the designed blueprint), and 4) constitution of part of a non-robust factor (i.e., a factor with < 3 items; [26, 29, 31–33]. Subsequently, 8 items were removed, resulting in a final set of 25 items.

In a final EFA of the 25 items (see Table 1), six factors had eigenvalues greater than 1 and explained 73.4% of the variance in the items. The final items, factor loadings, and scale statistics are presented in Table 1. The factors were labelled; Sleep Maintenance Problems (4 items), Co-Sleeping Behaviours (4 items), Bedtime Routines (5 items), Bedtime Resistance (5 items), Bedtime Worries (3 items) and Bedtime Fears (4 items). Factor 1, Sleep Maintenance Problems, had an eigenvalue of 8.16 and accounted for 32.65% of the variance in the items. The four items loading highly onto this factor reflected the extent to which children have difficulty putting themselves back to sleep after waking and had loadings that ranged from 0.83 to 0.51. Factor 2, Co-Sleeping Behaviours, contained four items reflecting the extent to which parents and children engage in co-sleeping at night, with loadings ranging from 0.95 to 0.72. The five items loading onto Factor 3, Bedtime Routines, ranged from 0.85 to 0.55 and reflected the extent to which families consistently engage in a bedtime routine. Factor 4, Bedtime Resistance, contained five items reflecting the extent to which children exhibit oppositional behaviour at bedtime and resist the bedtime process, with item loadings ranging from 0.84 to 0.69. The three items loading onto Factor 5, Bedtime Worries, reflected the extent to which children’s worries interfere with sleep, with loadings ranging from 0.87 to 0.69. Finally, Factor 6, Bedtime Fears, contained four items reflecting the extent to which children demonstrate night-time related fears, with loadings ranging from 0.86 to 0.61. All factors were significantly correlated with each other (*r*’s ranged from 0.11 to 0.52). Cronbach’s alphas were acceptable to high for all subscales (α’s of 0.88, 0.93, 0.82, 0.88, 0.85, and 0.86 respectively for Factors 1–6) and Cronbach’s alpha for the total composite score was high (α = 0.91).

## Study 2

The aims of Study 2 were to confirm the factor structure of the 25-item MAVBICS (Section 2) developed in Study 1, investigate the possibility of a higher order factor structure, and assess convergent validity through correlations with existing measures of behavioural sleep problems, anxiety and externalising behaviours. It was hypothesised that: (1) the factor structure of the MAVBICS would be confirmed; (2) that there would be positive associations between the MAVBICS Bedtime Fears subscale, the MAVBICS Bedtime Worries subscale, and measures of child anxiety, sleep anxiety, bedtime resistance and night waking; (3) that there would be positive associations between the MAVBICS Co-Sleeping Behaviours subscale and measures of child anxiety and sleep anxiety; (4) that there would be positive associations between the MAVBICS Bedtime Resistance subscale and measures of child anxiety, sleep anxiety, bedtime resistance and externalizing behaviour and; (5) that there would be positive associations between the MAVBICS Bedtime Routine subscale, the MAVBICS Sleep Maintenance Problems subscale, and a measure of night waking.

## Study 2 Method

### Participants

The participants were 313 male (4.2%) and female (94.9%) primary caregivers aged between 21 and 55 years (M = 36.22 *SD* = 5.40), who reported being either the mother (93%), father (3.5%), grandparent (0.6%), relative (1.9%) or other caregiver (1%) of a child aged between 3 and 12 years old. Another 99 participants began the questionnaire but were excluded because they did not indicate consent to their data being used for the study, or did not go on to begin the questionnaire after signing up. Detailed demographic data for the sample is provided in Supplementary Tables 1 and 2. Of the participants in the sample, 14.4% were university students from various campuses within Queensland Australia, 31.3% (majority) reported a bachelor’s degree as their highest level of education, 41.9% reported a household income of between $100,000–$2000,000 a year, 43.5% were employed casually or part time, and 86.6% were Australian Caucasian. Of the participants in the sample, 46.3% reported having a child aged between 3–5 years. Most participants (93.3%) reported having a child without any formal mental health, sleep or developmental diagnosis. Of the sample, 33.5%, 16% and 4.2% of the sample believed that their child had a mild, moderate and severe sleep problem respectively.

### Measures

#### Caregiver Demographics, Child Demographics and Parent-Reported Sleep Problems

Child and parent demographic items, and the parent-reported sleep problems item, were identical to those used in Study 1.

*Child Sleep Habits Questionnaire* (CSHQ; [22]. The CSHQ is a 33-item, parent-report measure designed to assess common sleep behaviours in children aged 4 to 12 years. Respondents are required to rate the frequency with which each item has occurred in the last week on a three-point Likert scale from 1 (rarely; 0–1 night per week) through 2 (sometimes; 2–4 nights per week) to 3 (usually; 5–7 nights per week). Eight subscales relating to Bedtime Resistance, Sleep Onset Delay (1 item), Sleep Duration, Sleep Anxiety, Night Wakings, Parasomnias, Daytime Sleepiness and Sleep Disordered Breathing, may be derived. The total CSHQ ranges from 33 to 99, with total scores over 41 indicative of a clinical sleep problem [22]. In addition to the total score, the current study utilised the Bedtime Resistance (6 items), Sleep Anxiety (4 items) and Night Waking (3 items) subscales. The scale has been used with parent samples of children aged 2–12 years [34], it meets the minimal levels for acceptable reliability in community samples (α = 0.68), and it has demonstrated adequate reliability in clinical sleep disordered samples (α = 0.78; [22]. The internal consistency for the total composite score in the present study was good (α = 0.84). The internal consistencies of the bedtime resistance, sleep anxiety and night waking subscales were variable in the current study, with α = 0.77, α = 0.64, and α = 0.71 respectively.

#### Anxiety

Two age-appropriate anxiety measures were used: The Preschool Anxiety Scale (PAS; [35] and the Spence Children’s Anxiety Scale- Parent (SCAS-P; [36]. The PAS is a 28-item parent rated scale designed to assess anxiety in children aged 3–6 years and was completed by participants with a child aged 3–6 years. Respondents are required to rate on a 5-point scale from 0 (not at all true) to 4 (very often true), the frequency with which each item is true for their child. Items are summed to produce a total score that may range from 0–28, with higher scores reflecting higher anxiety. The PAS has demonstrated strong psychometric properties and excellent internal consistency in prior studies [35]. The internal consistency of the total SCAS composite in the present study was excellent (α = 0.92).

The SCAS-P is a 38-item parent report scale developed to assess anxiety symptoms in children aged 7–18 years, and was completed by participants with a child aged 7–12 years. Respondents are required to rate the frequency with which each item applies to their child on a scale from 0 (never) to 3 (always). Items are summed to produce a total score that may range from 0–114, with higher scores reflecting higher levels of anxiety. The SCAS total score has demonstrated high internal consistency in previous studies (α = 0.89; [36] and excellent internal consistency in the present study (α = 0.92).

*Conduct Problems subscale of the Strengths and Difficulties Questionnaire* (SDQ; [37]. The Conduct subscale of the SDQ is a 5-item, parent-rated behavioural screening questionnaire for young people aged 2–17 years. It consists of two versions, one for children aged 2–4 years, and one for children aged 5–17 years. The two versions are consistent, although the wording on several items differs to ensure age appropriateness (e.g., often argumentative with adults [young child] versus often lies or cheats [child]). Items are scored from 0 (not true), through 1 (somewhat true), to 2 (certainly true), with subscale scores therefore ranging from 0–10, and higher scores indicating greater behaviour problems. The SDQ has displayed good psychometric properties in previous research [38]. In the current sample, the conduct subscale showed good internal consistency across both younger (α = 0.72) and older (α = 0.68) child samples.

### Procedure

The procedure of Study 2 was identical to that of Study 1.

#### Overview of Study 2 Data Analyses

AMOS v25 [39] was used to conduct a confirmatory factor analysis (CFA) using maximum likelihood estimation. Model fit was determined using χ^2^, root mean square error of association (RMSEA), the standardized root-mean-square residual (SRMR), the comparative fit index (CFI) and the Tucker–Lewis Index (TLI). Guidelines suggest that a non-significant χ^2^ is indicative of good model fit. However, this statistic is known to be sensitive to sample size, meaning that even trivial deviations from a perfect model are often statistically significant in samples as large as that used in this study. Bollen [40] therefore recommends dividing the χ^2^ by the degrees of freedom, with ratios of 2–3 representing good fit. RMSEA values below 0.05 are considered good, values between 0.05 and 0.08 are considered indicative of fair fit, and values between 0.08 and 0.10 are considered an indication of mediocre fit [41, 42]. SRMR values of < 0.08 indicate good model fit [43]. CFI and TLI values > 0.90 indicate acceptable model fit, and values > 0.95 indicate good model fit [42]. Internal consistency was assessed using Cronbach’s α. Convergent and discriminant validity were tested using bivariate correlations between the MAVBICS, the CSHQ subscales, the SDQ, the PAS and the SCAS-P.

## Study 2 Results

Prior to analyses, descriptive statistics were checked for skew and outliers. The results revealed 10 univariate outliers greater than 3 standard deviations from the mean. These participants were excluded from further analyses, leaving a final sample of 303. The CFA was performed with all items allowed to load only onto their respective factors, and factors allowed to covary as per the EFA results and a-priori theory. Although the CFA produced a significant χ^2^ (260, *N* = 303) = 635.53 *p* < 0.001, the ratio of χ^2^ to df was 2.4 indicating a good fit of the model to the data [40]. Other fit indices indicated that the CFA had an acceptable to good fit to the data (CFI = 0.92, IFI = 0.92, TLI = 0.91, RMSEA = 0.06, SRMR = 0.05; [44, 41, 42]. Table 1 presents the standardized confirmatory factor loadings for each item, as well as the Cronbach’s α for each subscale. As shown in Table 2, the correlations between each of the MAVBICS subscales ranged from weak to moderate (*r* = 0.09–0.55).

In accordance with apriori theory, a second higher order model was tested, with the results also revealing good model fit (CFI = 0.91, IFI = 0.91, TLI = 0.90, RMSEA = 0.07, SRMR = 0.07. The loadings on the second order ‘sleep problems’ factor were Bedtime Resistance (0.57), Co-Sleeping Behaviours (0.53) Bedtime Fears (0.69), Bedtime Routines (0.20), Bedtime Worries (0.54) and Sleep Maintenance Problems (0.73). In addition, a general factor model was fit, whereby all 25 items were allowed to load onto a single sleep problems factor. This model had a poor fit to the data, χ^2^ (275, *N* = 303) = 3435.26, *p* < 0.001. RMSEA = 0.19, CFI = 0.34, TLI = 0.28, IFI = 0.35, SRMR = 0.16. Item loadings were mostly adequate, ranging from 0.15–0.61.

*Convergent validity.* Table 2 reports the bivariate correlations between the MAVBICS total score and subscale scores, and the convergent validity variables (i.e., the CSHQ-Total, CSHQ- Bedtime Resistance, CSHQ-Sleep Duration, CSHQ-Sleep Anxiety, CSHQ-Night Wakings, SCAS, PAS, and the SDQ). The majority of predicted correlations were significant, in the predicted directions, and were of moderate to high strength. The exception to this more general rule was the Bedtime Routines subscale, that was not found to correlate with a measure of night waking (and was not predicted to do so).

## Study 3

The aim of Study 3 was to investigate the temporal stability of the MAVBICS over a 2-week period.

## Study 3 Method

### Participants

The participants were 53 mothers aged between 24 and 46 years (*M* = 36.77, *SD* = 4.45), of a child aged between 3 and 12 years. Of the participants in this sample, 84.9% were employed (45.3% part-time, 39.6% fulltime), 41.5% reported a bachelor degree as their highest level of education, 60.4% reported a household income of between $100,001 and $200,000, 73.6% were married, and 96.2% were Caucasian Australian. Of the participants in the sample, 56.6% reported having a child aged 3–5 years, 34.0% believed that their child had mild sleep problems, and 13.2% believed that their child had moderate or severe sleep problems.

### Measures

*Caregiver Demographics, Child Demographics and Parent-Reported Sleep Problems*. Parent and child demographic questions, as well as the parent-reported sleep problem item, were identical to those used in Studies 1 and 2.

*Child Insomnia Problems*. The Manifestations and Vulnerabilities for Behavioural Insomnia in Childhood Scale (MAVBICS) was used.

### Procedure

The same procedure used in Studies 1 and 2 was used in Study 3, with some minor exceptions. At the end of the Study 2 survey, participants were given the option to consent (via radio buttons) to participate in a short follow up survey in exchange for further course credit, or an extra nomination into the prize draw. The 100 participants who consented were instructed to create a personal code (consisting of a colour and animal e.g., rainbow otter) and enter it into a text box. They were then directed to a new survey site where they re-entered their code and email address so that their data could remain anonymous while being linked across the two time points. In the lead up to the follow-up assessment, participants were reminded (via email) several times to again complete the MAVBICS, and were sent a link to complete the survey within the allocated time frame (i.e., 48 h ± 2 weeks from study 2 completion). In the email body, participants were reminded of their unique code and instructed to enter it at the beginning of the Study 3 survey. Of the 100 participants who consented, 53 completed the follow up survey on time. Only participants who completed the repeated assessment in the two-week retest period were included in the analysis.

## Results

At Time 1, Cronbach’s α ranged from 0.88 to 0.91 for the 6 subscales of the MAVBICS and was 0.91 for the composite MAVBICS score. At Time 2, Cronbach's α ranged from 0.85 to 0.91 for the 6 subscales of the MAVBICS and was 0.91 for the composite MAVBICS score. All 2-week test–retest reliability coefficients were strong, with *r*'s ranging from a low of 0.84 (*p* < 0.01) for the MAVBICS Bedtime Resistance subscale to a high of 0.95 (*p* < 0.01) for the MAVBICS Co-Sleeping Behaviours subscale.

## Discussion

The goal of this series of studies was to develop and test the psychometric properties of a comprehensive parent-reported assessment measuring the manifestations of, and factors underpinning, behavioural insomnia in children; the Manifestations and Vulnerabilities to Behavioural Insomnia in Childhood Scale (MAVBICS). The first section of the MAVBICS (Section 1) assesses information on general sleep and bedtime behaviours. The second section of the MAVBICS (Section 2) was the focus of this series of three studies, and was designed to assess six theoretically derived domains, which, from a cognitive-behavioural perspective, represent manifestations of, and / or are thought to underly and contribute to, sleep problems in children: Bedtime Routines, Bedtime Fears, Bedtime Worries, Bedtime Resistance, Sleep Maintenance Problems, and Co-Sleeping Behaviours. From a CBT perspective, popular scales purporting to assess childhood sleep problems suffer conceptual drawbacks, including reliance on singular items to assess behavioural sleep issues (such as bedtime routines, and co-sleeping behaviour) or affective issues (such as night-time anxiety), or a predominant focus on medically related sleep problems (such as disordered breathing and parasomnias). Many of these scales also suffer from limited psychometric testing or display poor item statistics), limiting the usefulness of such measures for both research and clinical purposes (for a review, see [16, 19]. The MAVBICS was designed to address these gaps in the literature, through the development of a theory derived multidimensional measure of childhood behavioural insomnia problems and vulnerabilities, the items of which assess a range of parent and child factors that may manifest as, and/or underlie, behavioural insomnia in children aged 3–12 years. A copy of the full MAVBICS (Sections 1 and 2) is freely available in the Supplementary materials.

With minor exceptions, findings from the 3 component studies were consistent with hypotheses. The results suggested that the 25 component items of Section 2 of the MAVBICS loaded highly onto the six hypothesised factors (Bedtime Routine, Bedtime Fears, Bedtime Worries, Bedtime Resistance, Sleep Maintenance Problems, and Co-Sleeping Behaviours), that the reliabilities of the subscales and total score were high, that the total score and subscales correlated with associated measures in ways that supported convergent validity, and that the temporal stability of the instrument over a two-week period was high. Together, the results suggest that (with the exception of the Bedtime Routines subscale that is discussed in detail below), the MAVBICS subscales provide valid assessments of important parent and child factors that may manifest and/or underlie behavioural insomnia in childhood, and that the MAVBICS total score may have some utility as an indicator of a child’s vulnerability to behavioural insomnia. These results are first summarised and then discussed further in the paragraphs below.

Studies 1 and 2 respectively established and confirmed the factor structure of Section 2 of the MAVBICS, revealing six distinct and mostly correlated factors that aligned with theory to assess a variety of domains that contribute to behavioural insomnia in children. The first factor, Sleep Maintenance Problems, assessed the extent to which children have problems returning to sleep after waking through the night. The second factor, Co-Sleeping Behaviours, assessed the extent of child and parent co-sleeping behaviour. The third factor, Bedtime Routines, assessed the extent to which there is an established and consistently implemented bedtime routine within the household. The fourth factor, Bedtime Resistance, assessed the level of oppositional behaviour at bedtime, or behaviour designed to resist or delay bed and/or sleep time. The fifth factor, Bedtime Worries, assessed the extent to which children experience worry at night that interferes with sleep. The sixth factor, Bedtime Fears, assessed the degree to which night-time related fears disrupt sleep. Each of the MAVBICS subscales demonstrated positive associations with one another in Studies 1 and 2, with the exception of Bedtime Routines, which correlated only with the Co-Sleeping Behaviours subscale in Study 2. All subscales were found to correlate with the total score, and all scores including the total score, demonstrated strong internal consistencies. Finally, all MAVBICS subscales and the total score were found to predict child sleep problems (i.e., the CSHQ- total score). Together, these results provide strong preliminary evidence for the validity of the MAVBICS subscale scores as manifestations of, and factors underpinning, behavioural insomnia problems in children. Importantly, the results are consistent with the definition of Behavioural Insomnia in Childhood listed by the International Classification for Sleep Disorders (ISCD-3), which stresses the importance of considering factors pertaining to both parent (e.g., limit setting through routine, or the creation of negative sleep associations such as co-sleeping) and child (e.g., resistant behaviour, sleep onset/maintenance problems due to anxiety, or problematic sleep associations such as co-sleeping), in the assessment of behavioural insomnia in childhood [1].

While the abovementioned findings support the overall validity of the MAVBICS subscales, there are some important limiting features to consider and acknowledge. Importantly, the results suggest that the MAVBICS Bedtime Routines subscale may be less useful as an indicator of sleep difficulties in children compared to the other MAVBICS subscales. In particular, the results of Study 2 found that the Bedtime Routines subscale did not correlate with any of the other MAVBICS subscales except for Co-Sleeping Behaviours, and while the Bedtime Routines subscale was found to predict sleep problems, the association was only weak. These are unexpected findings given extant literature demonstrating the importance of bedtime routines for optimising sleep [45]. One explanation may lie in the wording of the subscale items. The MAVBICS items assess presence of a well implemented bedtime routine (e.g., ‘In our household, there is a set order of activities before bed’) and are then reverse scored to indicate potential problems. Previous literature in scale development has found that reverse-scored items are not always indicative of the opposite construct [46]. Indeed, a household may have a set order of activities before bed, but these activities may not be conducive to sleep (e.g., high energy play). That is, there may be a set order, and parents may implement the routine consistently, but the routine itself might not be optimal. Thus, a direct assessment of ‘problems’ with, and quality of, the bedtime routine would likely improve representation of this construct. Future research should test this hypothesis.

A further aim of Study 2 was to establish the construct validity of the MAVBICS subscales. The pattern of correlations observed between the majority of MAVBICS subscales and the constructs they were hypothesised to relate to, were theoretically and intuitively consistent. Extant literature suggests that anxiety in paediatric populations can detrimentally affect sleep quality [45, 47], and CBT theory and research suggests that paediatric anxiety is often associated with bedtime resistant behaviour and parental accommodation behaviour such as co-sleeping [9, 45, 48]. In keeping with this prior research, the MAVBICS subscales of Bedtime Fears, Bedtime Worries, Co-Sleeping Behaviours and Bedtime Resistance were all associated with higher levels of child anxiety (as measured by the PAS and SCAS) and CSHQ sleep anxiety. The MAVBICS Bedtime Fears and MAVBICS Bedtime Worries scales also predicted higher CSHQ bedtime resistance and CSHQ night waking, while MAVBICS Bedtime Resistance was associated with CSHQ bedtime resistance and externalising behaviour (as measured by the SDQ). Finally, while the MAVBICS sleep Maintenance Problems scale was positively associated with CSHQ night waking, the MAVBICS Bedtime Routine subscale was not. This finding is contrary to considerable existing research indicating that behavioural interventions such as bedtime routines can improve overall sleep quality and reduce night awakenings [6, 49], and provides further evidence that the Bedtime Routines subscale has limitations that need to be addressed. However, the Bedtime Routines subscale aside, the results of Studies 1 and 2 highlight that the MAVBICS subscale scores correlate with each other, and with theoretically relevant constructs in ways that suggest they are distinct and valid domains of the parent–child factors underlying behavioral sleep problems in children.

Finally, given the research that sleep problems in childhood are unlikely to remit without intervention [3], MAVBICS scores were expected to demonstrate test–retest reliability, with moderate temporal stability over a 2-week period. The results from Study 3 supported this hypothesis, suggesting that the MAVBICS scores are consistent over time.

### Limitations and Directions for Future Research

The findings surrounding the Bedtime Routines subscale points to a potential limitation in the operationalization of this domain that should be addressed in future research. Additionally, the sensitivity and specificity of the MAVBICS was not tested in this series of studies, and therefore future research should ascertain the ability of Section 2 of the MAVBICS to discriminate between those with sleep problems and those without. Additionally, future research may wish to investigate any meaningful developmental differences between children of different ages by comparing the MAVBICS subscale means between age groups, and by generating normative data accordingly. Finally, the sample used in the current research was relatively homogenous. Future research with the MAVBICS would benefit from ensuring the recruitment of families from more diverse ethnic and socio-demographic backgrounds, as well as the recruitment of more fathers.

## Conclusions

The collective findings of this research support the supposition that subscale scores on the MAVBICS constitute valid indicators of the various parent and child related factors that may manifest as, and/or underpin, behavioural insomnia in children. Filling an important gap in the literature, the MAVBICS delivers both a theoretically derived, and psychometrically evaluated multidimensional assessment of six distinct domains: Sleep Maintenance Problems, Co-sleeping Behaviours, Bedtime Resistance, Bedtime Worries, Bedtime Fears, and Bedtime Routines. Strong support for the construct and content validity of scores on the first five factors was garnered over 3 different studies, with results offering partial support for the validity of scores on the Bedtime Routine factor and a total score. To our knowledge, there are no existing measures that provide both a comprehensive and valid assessment of the manifestations of, and factors underlying, behavioural sleep problems in paediatric populations, making the MAVBICS the only assessment of its kind. While further adjustments and validation research is required, preliminary results point to the usefulness of the MAVBICS in identifying vulnerability to behavioural insomnia problems among children and the mechanisms that may contribute to and / or maintain these issues. The MAVBICS is therefore likely to have significant empirical and clinical value.

## Summary

This series of three studies outlines the development and psychometric evaluation of the Manifestations and Vulnerabilities of Behavioural Insomnia in Childhood Scale (MAVBICS). The exploratory factor analysis in Study 1 suggested that the 25 MAVBICS items loaded onto 6 factors; Sleep Maintenance Problems, Co-Sleeping Behaviours, Bedtime Routines, Bedtime Resistance, Bedtime Worries, and Bedtime Fears. In Study 2, this factor structure was confirmed and convergent validity was established between MAVBICS scores and other sleep, anxiety and behaviour measures. Finally, the test-retest reliability of the MAVBICS over a 2-week period was found to be strong. Although the Bedtime Routines subscale requires further investigation and potentially modification, overall the MAVBICS total score and subscales were found to be reliable.

## Supplementary Information

Below is the link to the electronic supplementary material.

Supplementary file1 (DOCX 22 KB)

Supplementary file2 (DOCX 23 KB)

## Data Availability

Not applicable.
