# Supplementary material for: Helping Clinicians Conceptualise Behavioural Insomnia in Children: Development of the Manifestations and Vulnerabilities of Behavioural Insomnia in Childhood Scale (MAVBICS)
Source: Child Psychiatry Hum Dev. 2023 Oct 5;56(4):907–22. doi: 10.1007/s10578-023-01606-w (PMC12289719; doi:10.1007/s10578-023-01606-w)
Supplement: Supplementary file 1 — Supplementary file1 (DOCX 22 KB) [file 10578_2023_1606_MOESM1_ESM.docx]

Table 1

Caregiver Demographic Details for the Exploratory and Confirmatory Factor Analyses

|  | Study Number | |
| --- | --- | --- |
| Demographic Variables | 1 – EFA  (*N* = 328) | 2 – CFA  (*N* = 313) |
| Gender |  |  |
| Female | 315 (96%) | 297 (94.9%) |
| Male | 11 (3.4%) | 13 (4.2%) |
| Other | 2 (0.6%) | 3 (0.9%) |
| Age |  |  |
| 18-35 years | 145 (44.2%) | 137 (43.8%) |
| 36-45 years | 163 (49.7%) | 165 (52.7%) |
| 46 years + | 20 (0.06%) | 11 (3.5%%) |
| Ethnicity |  |  |
| Caucasian | 295 (89.9%) | 271 (86.6%) |
| Aboriginal /TSI | 4 (1.2%) | 6 (1.6%) |
| Asian | 17 (5.2%) | 20 (6.4%) |
| Other | 12 (3.7%) | 16 (5.1%) |
| Relationship to the Child |  |  |
| Mother | 313 (95.4%) | 291 (93%) |
| Father | 9 (2.7%) | 11 (3.5%) |
| Grandparent | 1 (0.3%) | 2 (0.6%) |
| Other relative | 4 (1.2%) | 6 (1.9%) |
| Caregiver | 1 (0.3%) | 3 (1.0%) |
| Relationship Status |  |  |
| Single | 15 (4.6%) | 25 (8%) |
| Defacto | 47 (14.3%) | 41 (13.1%) |
| Married | 238 (72.6%) | 219 (70%) |
| Separated/Divorced | 22 (6.7%) | 25 (8.0%) |
| Other | 6 (1.8%) | 3 (0.9%) |
| Employment Status |  |  |
| Student | 35 (10.7%) | 45 (14.4%) |
| Full time | 105 (32%) | 88 (28.1%) |
| Part time | 152 (46.3%) | 136 (43.5%) |
| Unemployed | 33 (10.1%) | 36 (11.5%) |
| Other | 3 (0.9%) | 8 (2.5%) |
| Highest level of Education |  |  |
| Below grade 10 | 2 (0.6%) | 2 (0.6%) |
| Grade 10 -11 | 15 (4.6%) | 8 (2.6%) |
| Grade 12 | 47 (14.3%) | 40 (12.8%) |
| TAFE degree | 80 (24.4%) | 90 (28.8%) |
| Bachelor degree | 122 (37.2%) | 98 (31.3%) |
| Postgraduate degree | 62 (18.9%) | 75 (24.0%) |
| Parenting Status |  |  |
| Single parent | 31 (9.5%) | 28 (8.9%) |
| Dual parent living with child | 276 (84.1%) | 255 (81.5%) |
| Co-parenting with shared custody | 16 (4.1%) | 19 (6.1%) |
| Other | 5 (1.5%) | 11 (3.5%) |
| Annual Household Income |  |  |
| $0 – 40k | 3 (7.0%) | 15 (4.8%) |
| $40, 001 – 60k | 17 (5.2%) | 23 (7.3%) |
| $60, 001 – 80k | 27 (8.2%) | 35 (11.2%) |
| $80,001 – 100k | 52 (15.9%) | 47 (15%) |
| $100,001 – 200k | 151 (46%) | 131 (41.9%) |
| >$200k | 45 (13.7%) | 44 (14.1%) |
| No response | 13 (4%) | 18 (5.8%) |

Note. * EFA = exploratory factor analyses, CFA = confirmatory factor analyses, TSI = Torres Strait Islander. Study 3 utilised a subset of participants from study 2, please see text for demographics of this sample.

Table 2

Child Demographic Details for the Exploratory and Confirmatory Factor Analyses

|  | Study Number | |
| --- | --- | --- |
| Demographic Variables | 1 – EFA  (*N* = 328) | 2 – CFA  (N = 313) |
| Child’s Gender |  |  |
| Female | 142 (43.3%) | 145 (46.3%) |
| Male | 186 (56.7%) | 166 (53%) |
| Child’s Age |  |  |
| 3-5 years | 181 (55.2%) | 145 (46.3%) |
| 6- 12 years | 147 (44.8%) | 168 (53.7%) |
| Child’s Ethnicity |  |  |
| Caucasian | 284 (86.6%) | 267 (85.3%) |
| Aboriginal / TSI | 7 (2.1%) | 6 (1.9%) |
| Asian | 16 (4.9%) | 11 (3.5%) |
| Other | 21 (6.4%) | 29 (9.3%) |
| Child Resides in |  |  |
| One Residence | 309 (94.2%) | 285 (91.1%) |
| More than one residence | 19 (5.8%) | 28 (8.9%) |
| Parent Rated Sleep Problem |  |  |
| No problem | 196 (59.8%) | 145 (46.4%) |
| Mild | 99 (30.2%) | 105 (33.5%) |
| Moderate | 27 (8.2%) | 50 (16%) |
| Severe | 6 (1.8%) | 13 (4.2%) |
| Formal Diagnosis of a Sleep Problem |  |  |
| Yes | 3 (0.9%) | 4 (1.3%) |
| No | 325 (99.1%) | 309 (98.7%) |
| Formal Diagnosis – Other |  |  |
| Anxiety Disorder | 14 (4.2%) | 12 (3.8%) |
| Speech / Language Delay | 7 (2.1%) | 19 (6.1%) |
| Visual/ Hearing Difficulty | 5 (1.5%) | 13 (4.2%) |
| ADHD | 10 (3.0%) | 21 (6.7%) |
| ASD | 15 (4.2%) | 13 (4.2%) |
| Medical Issue/ Physical Disability | 4 (1.2%) | 15 (4.8%) |

Note. * TSI = Torres Strait Islander, ADHD = Attention Deficit Hyperactivity Disorder, ASD = autism spectrum disorder. De facto = relationship where people live together as an unmarried couple. Study 3 utilised a subset of participants from study 2, please see text for demographics of this sample.
