# Supplementary material for: Helping Clinicians Conceptualise Behavioural Insomnia in Children: Development of the Manifestations and Vulnerabilities of Behavioural Insomnia in Childhood Scale (MAVBICS)
Source: Child Psychiatry Hum Dev. 2023 Oct 5;56(4):907–22. doi: 10.1007/s10578-023-01606-w (PMC12289719; doi:10.1007/s10578-023-01606-w)
Supplement: Supplementary file 2 — Supplementary file2 (DOCX 23 KB) [file 10578_2023_1606_MOESM2_ESM.docx]

Manifestations and Vulnerabilities of Behavioural Insomnia in Childhood Scale (MAVBICS)

Would you describe your child as having a sleep problem, and if so how severe would you describe it as being?

O No Problem O Mild O Moderate O Severe

What is the usual time your child wakes up on weekdays? ___________________________

What is the usual time your child wakes up on weekends? ___________________________

What time does your child usually go to bed on weekdays? (this means the time your child is in bed with the lights out, not what time they fall asleep) ___________________________

What time does your child usually go to sleep on weekdays? ___________________________

What time does your child usually go to bed on weekends? (this means the time your child is in bed with the lights out, not what time they fall asleep) ___________________________

What time does your child usually go to sleep on weekends? ___________________________

How many times does your child wake during the night? ___________________________

If you child does wake during the night, what is the total amount of time your child is awake during the night? ___________________________

How many times does your child nap during the day? ___________________________

What is the total amount of time that your child naps during the day? ___________________________

Now we would like to ask some questions about your child's sleep and bedtime related behaviours. Thinking about the past week, please rate how true the following statements are about your child's sleep and bedtime behaviour. If the last week was unusual (e.g. your child was unwell or there was a holiday period), respond to the statements based on a typical week.

Please think about the following statements in relation to your child and household, and rate how true each of the following statements are.

|  |  | Never True | Rarely true | Sometimes True | Often True | Very Often True | Always True |
| --- | --- | --- | --- | --- | --- | --- | --- |
| 1 | My child wakes in the night with panic or fear from a nightmare / night terror |  |  |  |  |  |  |
| 2 | My child has nightmares |  |  |  |  |  |  |
| 3 | My child wets the bed |  |  |  |  |  |  |
| 4 | My child snores loudly |  |  |  |  |  |  |
| 5 | My child stops breathing / snorts / gasps in their sleep |  |  |  |  |  |  |
| 6 | My child sleepwalks |  |  |  |  |  |  |
| 7 | My child takes longer than 20 minutes to fall asleep |  |  |  |  |  |  |

Manifestations and Vulnerabilities of Behavioural Insomnia in Childhood Scale (MAVBICS)

Below is a series of statements that describe a range of bedtime and sleep related behaviours your child might demonstrate. We would like you to think about your child and their behaviour over the past week when responding to the questions below. If there was a reason that the last week was unusual (such as your child was unwell or there was a holiday period), respond to the questions based on a typical week.

Please think about the following statements in relation to your child and household, and rate how true each of the following statements are.

|  |  | Never True | Rarely true | Sometimes True | Often True | Very Often True | Always True |
| --- | --- | --- | --- | --- | --- | --- | --- |
| 1 | My child sleeps through the whole night |  |  |  |  |  |  |
| 2 | My child wakes up during the night and wakes another family member |  |  |  |  |  |  |
| 3 | After falling asleep, my child wakes and calls out |  |  |  |  |  |  |
| 4 | My child repeatedly gets out of their bed at night time |  |  |  |  |  |  |
| 5 | At night, my child sleeps in the same bed as me, or another family member |  |  |  |  |  |  |
| 6 | My child sleeps alone in their bed throughout the night |  |  |  |  |  |  |
| 7 | My child refuses to sleep in their own room by themselves |  |  |  |  |  |  |
| 8 | My child falls asleep, by themselves, in their own bed |  |  |  |  |  |  |
| 9 | In our household, there is a set order of activities before bed |  |  |  |  |  |  |
| 10 | In our household, all caregivers follow the same bedtime routine with my child |  |  |  |  |  |  |
| 11 | In our household, the activities before my child’s bedtime are the same each night |  |  |  |  |  |  |
| 12 | My child knows the activities they need to do before bed |  |  |  |  |  |  |
| 13 | I attempt to put my child to bed at the same time each night (within 15 minutes) |  |  |  |  |  |  |
| 14 | My child does things to delay going to bed |  |  |  |  |  |  |
| 15 | My child does not follow instructions bedtime |  |  |  |  |  |  |
| 16 | My child tantrums when asked to do things at bedtime |  |  |  |  |  |  |
| 17 | My child does not want to go to bed |  |  |  |  |  |  |
| 18 | At bedtime, my child makes repeated requests (e.g., for a drink, for food, for a cuddle) |  |  |  |  |  |  |
| 19 | My child worries about many different things when in bed |  |  |  |  |  |  |
| 20 | My child lies awake at night worrying |  |  |  |  |  |  |
| 21 | My child finds it difficult to sleep because they can't stop thinking |  |  |  |  |  |  |
| 22 | My child is scared of monsters, witches, or other scary things at night time |  |  |  |  |  |  |
| 23 | My child gets scared at bed / night time |  |  |  |  |  |  |
| 24 | My child is frightened of the dark |  |  |  |  |  |  |
| 25 | My child needs someone to tell them it's OK at night (e.g., about the dark, being alone, safety, monsters) |  |  |  |  |  |  |

Copyright Donovan, Uhlmann and Shiels (2023).
